# Supplementary material for: ProteinShader: illustrative rendering of macromolecules
Source: BMC Struct Biol. 2009 Mar 30;9:19. doi: 10.1186/1472-6807-9-19 (PMC2672931; doi:10.1186/1472-6807-9-19)
Supplement: Additional file 1 — ProteinShader program without source code. This compressed file contains the complete ProteinShader program including associated libraries, but no source code. A README.txt file gives an overview of the ProteinShader distribution, and the index.html file in the help subdirectory has directions on getting started with the program as well as a set of tutorials. [file 1472-6807-9-19-S1.zip › ProteinShader-beta-0_9_4-binary/help/api/org/proteinshader/graphics/shader/class-use/ShaderProgram.html]

Uses of Class org.proteinshader.graphics.shader.ShaderProgram (ProteinShader API)


|  |  |  |
| --- | --- | --- |
| **Overview** \| **Package** \| **Class** \| **Use** \| **Tree** \| **Deprecated** \| **Index** \| **Help** | |  |
| PREV   NEXT | **FRAMES**    **NO FRAMES**     **All Classes** |  |


---


## **Uses of Class org.proteinshader.graphics.shader.ShaderProgram**

| Packages that use ShaderProgram | |
| --- | --- |
| **org.proteinshader.graphics.shader** | Holds the classes needed for compiling and managing the vertex and fragment shaders used for texture mapping and custom lighting calculations. |

Uses of ShaderProgram in org.proteinshader.graphics.shader

| Methods in org.proteinshader.graphics.shader that return ShaderProgram | |
| --- | --- |
| `ShaderProgram` | `ShaderProgramFactory.compile(GL gl, ShaderEnum pair)`             Reads a pair of vertex and fragment shader files and then compiles and links them. |
| `ShaderProgram` | `ShaderProgramFactory.createShaderProgram(GL gl, File vertFile, File fragFile, ShaderEnum pair)`             Reads source code files for a vertex shader and a fragment shader and creates an OpenGL shader program object. |
| `ShaderProgram` | `ShaderProgramFactory.createShaderProgram(GL gl, String vertFilename, String fragFilename, ShaderEnum pair)`             Reads source code files for a vertex shader and a fragment shader and creates an OpenGL shader program object. |

| Methods in org.proteinshader.graphics.shader that return types with arguments of type ShaderProgram | |
| --- | --- |
| `List<ShaderProgram>` | `ShaderProgramFactory.compileAllShaders(GL gl)`             Compiles all shaders pairs defined by the ShaderEnum. |

| Methods in org.proteinshader.graphics.shader with parameters of type ShaderProgram | |
| --- | --- |
| `void` | `ShaderProgramFactory.deleteShaderProgram(GL gl, ShaderProgram program)`             Calls delete on each OpenGL shader and program object name held by the ShaderProgram given as an argument. |

---


|  |  |  |
| --- | --- | --- |
| **Overview** \| **Package** \| **Class** \| **Use** \| **Tree** \| **Deprecated** \| **Index** \| **Help** | |  |
| PREV   NEXT | **FRAMES**    **NO FRAMES**     **All Classes** |  |


---

# *Copyright © 2007-2008*
